# Iron-and-folic-acid supplementation among adolescents (aged 10–19 years) in two North Indian States, 2015–2016: a sex-stratified analysis

**DOI:** 10.1017/S136898002000508X

**Published:** 2022-03

**Authors:** Rajesh Kumar Rai

**Affiliations:** 1 Society for Health and Demographic Surveillance, Suri, West Bengal 731101, India; 2 Department of Global Health and Population, Harvard T H Chan School of Public Health, Boston, MA 02115, USA; 3 Department of Economics, University of Göttingen, Göttingen, Germany; 4 Centre for Modern Indian Studies, University of Göttingen, Göttingen, Germany

**Keywords:** Adolescent, Nutrition, Anaemia, Iron, India

## Abstract

**Objective:**

This study assessed the prevalence and predictors of receiving iron-and-folic-acid (IFA) supplement by male and female adolescents in two north Indian states.

**Design:**

The UDAYA (Understanding the lives of adolescents and young adults in Bihar and Uttar Pradesh) survey dataset was used. Conducted during 2015–2016, UDAYA was a state representative cross-sectional survey. To recruit sample, UDAYA adopted a multi-stage systematic sampling method with a household selection probability proportional to size. Weighted bivariate and multivariate logistic regression analyses were deployed. The variance inflation factor was estimated to check the presence of multicollinearity among variables included in regression model.

**Setting:**

The state of Bihar and Uttar Pradesh, India.

**Participants:**

A total of 10 433 individuals from Bihar and 10 161 individuals from Uttar Pradesh were included, totalling 20 594 individuals (male: 5969, female: 14 625) aged 10–19 years.

**Results:**

Overall, 3·6 % (95 % CI: 2·7, 4·7) of males and 4·8 % (95 % CI: 4·0, 5·7) of female adolescents received IFA supplement in preceding 1 year of survey date. Multivariate results indicate that IFA receipt varied with age, and state of residence among males, whereas religion and mother’s education were associated with IFA receipt among females. Irrespective of sex, adolescents living in rural areas had higher odds of receiving IFA supplement than adolescents in urban setting.

**Conclusions:**

Low coverage in receiving IFA supplement among adolescents is a serious concern for the success of anaemia reduction programme. While designing interventions for overall increase in IFA distribution, the socio-economic factors influencing IFA receipt must be considered.

Burden of undernutrition among adolescents (aged 10–19 years) has received insufficient attention by India’s public health researchers and programme and policy-makers. Among all interventions to address undernutrition including micronutrient deficiencies, strategies to address the burden of anaemia, especially Fe-deficiency anaemia, are often criticised^(1,2)^. Adolescents are considered as a high-risk group for anaemia and Fe deficiency as they have high requirements of Fe, poor dietary intake of Fe, high rate of infection and worm infestation, whereas the social norm of early marriage among girls and adolescent pregnancy could worsen their health condition^(3)^. If not treated, anaemia could cause delayed physical and psychomotor development, decreased physical capacity for physical exercise and productivity, and significant damage to central nervous system, and girls are disproportionately affected with deficit in intelligence quotient, impaired coordination of language and motor skills, poor attentiveness, memory and learning ability and lower school achievements^(2)^.

According to the Comprehensive National Nutrition Survey (CNNS) 2016–2018 report of India, 28·4 % of adolescents aged 10–19 years had some degree of anaemia and 41·5 % of anaemics were Fe-deficient^(4)^. CNNS report also highlighted the relationship between Fe deficiency and anaemia and estimated that 12 % of all adolescents were anaemic and Fe-deficient, 16·9 % were anaemic but not Fe-deficient, 9·5 % were Fe-deficient but not anaemic and 61·7 % of all adolescents neither had anaemia nor had Fe deficiency. To tackle the burden of Fe-deficiency anaemia, the Ministry of Health and Family Welfare (MoHFW) launched the National Iron Plus Initiative (NIPI) in 2011 in which adolescents were recommended to receive weekly consumption of 100 mg of elemental Fe and 500 mcg of folic acid (IFA)^(5)^. Thus, IFA intervention potentially corrects both Fe deficiency and Fe-deficiency anaemia. India covers both adolescent boys and adolescent girls for its IFA intervention because of an unacceptably high burden of anaemia among both sexes^(5)^. The adolescent beneficiaries are school-going children who will receive the prescribed dose through teachers, and out-of-school children will receive the dose through Anganwadi Centre (AWC) with some assistance from the Accredited Social Health Activists (ASHA)^(5)^. Anganwadi Workers (AWW) and ASHA are basic healthcare provider at the community level in India^(6)^.

Previous reports have successfully demonstrated the high burden of anaemia and low compliance of IFA consumption among adolescents and adult women^(1,4,5)^. A handful of empirical studies on IFA uptake among adolescents are available^(7,8)^, and those studies often lack population-based up-to-date analysis. Against this knowledge gap, this study analysed the 2015–2016 UDAYA (Understanding the lives of adolescents and young adults in Bihar and Uttar Pradesh) survey data to assess the prevalence and predictors of receiving IFA supplement by male and female adolescents aged 10–19 years. A supplementary descriptive analysis of IFA consumption was undertaken to elaborate the study findings. According to CNNS report, 28·1 % of all adolescents in Bihar and 31·6 % adolescents in Uttar Pradesh were diagnosed with anaemia, with 12·7 % adolescents in Bihar and 17·2 % in Uttar Pradesh were Fe-deficient. These alarming statistics call for understanding the barriers of receiving IFA supplement, which this study aimed to analyse.

## Methods

### Dataset

Dataset was retrieved from the UDAYA survey, a cross-sectional survey conducted by the Population Council (https://www.popcouncil.org/) during 2015–2016, covering adolescents aged 10–19 years^(9,10)^. Conducted in two north Indian states – Bihar (9) and Uttar Pradesh (10), estimates of UDAYA survey is state representative and used 2011 Census of India sampling frame. A total of 150 primary sampling units – 75 villages for rural respondents and 75 census wards for urban respondents in each state – were determined, followed by multi-stage systematic sampling design within each sampling domain was adopted. From the 2011 Census of India data, information on four variables, namely region, village/ward size, proportion of the population belonging to scheduled castes and scheduled tribes, and female literacy, were used to select households in rural and urban areas with selection probability proportional to size. In Bihar, 33 900 households were covered to select 10 433 respondents and Uttar Pradesh sampled 32 348 households and selected 10 161 respondents aged 10–19 years. Further details about sampling procedure of UDAYA survey could be retrieved from its published report^(9,10)^. For this study, responses from 10 433 individuals in Bihar and 10 161 individuals from Uttar Pradesh were included, totalling 20 594 adolescents (male: 5969, female: 14 625). No missing data were reported.

### Outcome event

In UDAYA survey, adolescents were asked if they had ‘received iron-and-folic-acid tablets and/or de-worming tablets in last one year from school or AWC or ASHA?’ Response against both iron-and-folic-acid (IFA) tablets and de-worming tablets were recorded separately. If the response was affirmative against the receipt of IFA, a subsequent question was posed – ‘In the last one month, how many times did you take iron-and-folic-acid tablets?’. Using response from the first question, this study tried to understand the prevalence and predictors of IFA receipt preceding 1 year of survey date, whereas information on consumption of IFA supplement during last month from survey date was also tabulated to understand how often adolescents consumed IFA.

### Predictor variable

Guided by the available literature on this issue, chosen predictor variables are age group, area of residence, religion, social group, adolescent’s education in years, mother’s years of education, wealth index and state. In subcategory of religion – ‘Islam/others’, over 99 % of adolescents were followers of Islam. By deploying principal component analysis^(11)^, wealth index was developed using household asset data on ownership of selected durable goods, including means of transportation, and data on access to a number of amenities. To understand the variation of IFA supplementation between states, a dummy variable representing both states (Bihar and Uttar Pradesh) was computed.

### Statistical approach

Bivariate analysis was conducted to understand the sample distribution and prevalence of IFA receipt and consumption among male and female adolescents in Bihar and Uttar Pradesh separately. Dataset from both Bihar and Uttar Pradesh was merged prior to running further analysis. Stratified by sex, unadjusted logistic regression was executed to assess the association between each predictor with IFA receipt, whereas multivariate logistic regression analysis adjusted for all potential predictors was conducted to understand the net effect of predictors on receiving IFA supplement by adolescents. Prior to running multivariate-adjusted logistic regression analysis, inclusion of predictors was checked for their multicollinearity by estimating variance inflation factor. The variance inflation factor against all predictors were less than 5 indicating low chance of multicollinearity (data not shown separately).

Appropriate sample weighting provided with the dataset was used to estimate the prevalence and to estimate the OR with 95 % CI from logistic regression. Instruction about the use of sample weighting was provided by the Population Council, and this study used the weighting for running analysis with both states (Bihar and Uttar Pradesh) combined. The statistical software – Stata version 14^(12)^ – was used to perform the statistical analysis, and statistical significance (*P*) of <0·05 (two-tailed) obtained from logistic regression was discussed. The ‘svy’ suite available with Stata software used for application of sample weighting which took care of complex survey design and provided robust estimates.

## Results

**Fig. 1:**
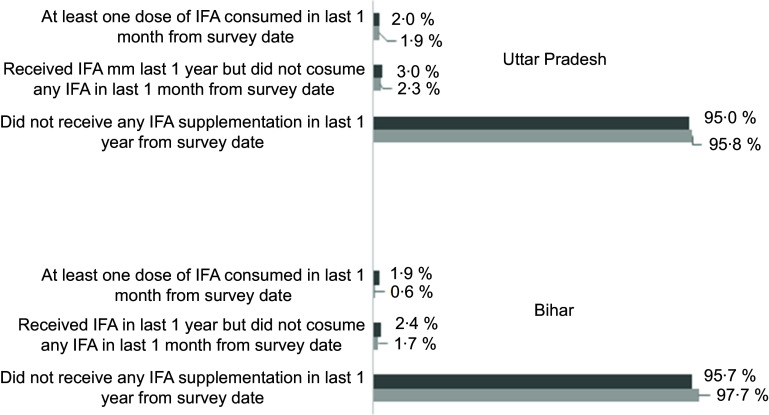
Percentage distribution of iron-and-folic-acid receipt and consumption among male and female adolescents in Uttar Pradesh and Bihar. IFA: iron-and-folic-acid. 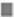, female; 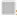, male

Table 1 represents sample distribution of male and female adolescents included in the analysis. Majority of adolescents belonged to 15–19 years age group and lived in rural area. Over 60 % of mothers of adolescents were uneducated. A sex-stratified analysis of prevalence and predictors of IFA receipt is presented in Table 2. Overall, 3·6 % (CI: 2·7, 4·7) of males and 4·8 % (CI: 4·0, 5·7) of females received IFA in last 1 year from the survey date. Multivariate logistic regression analysis adjusted for potential predictors reveal that males aged 15–19 years were less likely to receive IFA (OR: 0·39, CI: 0·22, 0·69, *P* = 0·001) as compared to adolescents aged 10–14 years. Irrespective of sex, adolescents living in rural area had higher odds of receiving IFA than adolescents from urban area. Unadjusted OR showed similar association for age group and area of residence. Female followers of Islam/other religion had lower odds of receiving IFA as compared to females who were followers of Hinduism (OR: 0·61, CI: 0·41, 0·92, *P* = 0·020). Mother’s with 1–9 years of education had increased likelihood of receiving IFA among female adolescents (OR: 1·60, CI: 1·13, 2·25, *P* = 0·008). Male adolescents from Uttar Pradesh were more likely (OR: 1·99, CI: 1·04, 3·82, *P* = 0·037) to receive IFA as compared to male adolescents from Bihar. Figure 1 attempted to understand the receipt and consumption of IFA among adolescents. Findings showed that only 1·9 % of females in Bihar and 2 % of females in Uttar Pradesh consumed at least one dose of IFA preceding 1 month of survey date, whereas only 0·6 % males had at least one dose of IFA supplement in Bihar which is lower than the IFA consumption among males in Uttar Pradesh (1·9 %).

**Table 1:** Sample distribution of adolescents in Bihar and Uttar Pradesh, stratified by sex

	Bihar		Uttar Pradesh	
	Male	Female	Male	Female
Age group				
10–14	35·7	10·1	34·2	12·7
15–19	64·3	89·9	65·8	87·3
Area of residence				
Urban	10·9	10·5	23·0	22·2
Rural	89·1	89·5	77·0	77·8
Religion				
Hinduism	85·9	85·9	82·2	74·1
Islam/others	14·1	14·1	17·8	25·9
Social group				
Others	12·9	10·9	21·5	23·6
OBC	64·3	64·5	50·1	50·5
SC/ST	22·8	24·7	28·4	25·8
Education (in years)				
0–9	81·0	76·0	71·1	63·4
≥10	19·0	24·1	28·9	36·7
Mother’s education (in years)				
Uneducated	77·2	77·3	67·3	72·3
1–9	14·6	15·5	20·4	18·0
≥10	8·2	7·2	12·3	9·6
Wealth index				
Poorest	8·8	12·1	12·8	13·8
Poorer	19·7	17·3	21·8	19·5
Middle	21·9	21·5	21·6	22·3
Richer	26·2	26·1	23·6	24·2
Richest	23·4	23·1	20·2	20·2
Total (unweighted)	2833	7600	3136	7025

OBC, other backward classes; SC, scheduled castes; ST, scheduled tribes.

* All estimates are weighted if not declared otherwise.

**Table 2:** Prevalence and predictors of receiving iron-and-folic-acid tablets in Bihar and Uttar Pradesh, stratified by sex*

	Male (*n* 5969)								Female (*n* 14 625)							
			Unadjusted model		*P*	Adjusted model		*P*	%	95 % CI	Unadjusted model		*P*	Adjusted model		*P*
	%	95 % CI	OR	95 % CI		OR	95 % CI				OR	95 % CI		OR	95 % CI	
Age group																
10–14	5·4	3·7–7·8	Referent			Referent			5·7	4·0–8·2	Referent			Referent		
15–19	2·6	1·9–3·6	0·48	0·30–0·75	0·002	0·39	0·22–0·69	0·001	4·7	3·9–5·6	0·80	0·55–1·16	0·235	0·81	0·56–1·18	0·278
Area of residence																
Urban	1·7	0·9–3·5	Referent			Referent			3·2	2·2–4·6	Referent			Referent		
Rural	4·0	3·0–5·3	2·33	1·08–5·03	0·031	2·72	1·22–6·05	0·014	5·1	4·2–6·2	1·64	1·06–2·53	0·025	1·84	1·16–2·92	0·010
Religion																
Hinduism	3·7	2·8–4·8	Referent			Referent			5·2	4·4–6·2	Referent			Referent		
Islam/others	3·0	1·4–6·5	0·81	0·38–1·74	0·585	0·88	0·40–1·95	0·755	3·2	2·1–4·8	0·59	0·39–0·89	0·013	0·61	0·41–0·92	0·020
Social group																
Others	4·6	3·1–6·9	Referent			Referent			5·2	3·7–7·3	Referent			Referent		
OBC	3·3	2·2–4·8	0·70	0·42–1·19	0·185	0·69	0·39–1·21	0·191	4·7	3·7–6·0	0·90	0·61–1·33	0·601	1·04	0·73–1·49	0·821
SC/ST	3·4	2·3–5·0	0·73	0·42–1·27	0·267	0·60	0·30–1·22	0·160	4·6	3·6–6·0	0·89	0·57–1·37	0·585	0·95	0·63–1·45	0·821
Education (in years)																
0–9	3·6	2·6–5·0	Referent			Referent			4·6	3·7–5·6	Referent			Referent		
≥10	3·4	2·3–5·2	0·94	0·58–1·54	0·81	1·60	0·91–2·82	0·103	5·2	4·2–6·4	1·14	0·90–1·44	0·290	0·97	0·75–1·26	0·815
Mother’s education (in years)																
Uneducated	3·7	2·7–5·1	Referent			Referent			4·2	3·3–5·2	Referent			Referent		
1–9	3·6	2·3–5·5	0·97	0·64–1·47	0·868	0·88	0·58–1·34	0·552	6·8	5·0–9·1	1·68	1·18–2·39	0·004	1·60	1·13–2·25	0·008
≥10	2·7	1·5–4·7	0·71	0·36–1·40	0·321	0·66	0·32–1·36	0·262	6·3	4·7–8·3	1·54	1·07–2·24	0·022	1·49	0·99–2·27	0·059
Wealth index																
Poorest	6·0	3·4–10·4	Referent			Referent			3·8	2·9–5·2	Referent			Referent		
Poorer	3·0	1·8–5·2	0·49	0·22–1·10	0·083	0·50	0·21–1·17	0·111	4·7	3·6–6·2	1·25	0·86–1·80	0·235	1·21	0·83–1·75	0·321
Middle	2·8	1·7–4·5	0·44	0·21–0·93	0·031	0·46	0·21–1·01	0·052	4·7	3·8–5·9	1·24	0·88–1·75	0·210	1·23	0·87–1·74	0·233
Richer	3·3	2·1–5·0	0·53	0·25–1·10	0·088	0·58	0·25–1·33	0·196	4·7	3·4–6·3	1·22	0·78–1·91	0·373	1·21	0·77–1·89	0·413
Richest	4·0	2·6–6·2	0·65	0·34–1·26	0·204	0·75	0·33–1·71	0·489	5·6	4·1–7·5	1·48	0·95–2·29	0·082	1·39	0·82–2·37	0·218
State																
Bihar	2·3	1·3–3·9	Referent			Referent			4·3	3·3–5·5	Referent			Referent		
Uttar Pradesh	4·2	3·0–5·7	1·85	0·97–3·54	0·061	1·99	1·04–3·82	0·037	5·0	4·0–6·3	1·19	0·84–1·70	0·329	1·25	0·88–1·78	0·211
Total	3·6	2·7–4·7							4·8	4·0–5·7						

OBC, other backward classes; SC, scheduled castes; ST, scheduled tribes; *P*, level of statistical significance.

* All estimates are weighted if not declared otherwise.

## Discussion

Using data from the 2015–16 UDAYA survey, this study aimed to examine prevalence and predictors of receiving IFA by adolescents aged 10–19 years in two north Indian states namely Bihar and Uttar Pradesh. In absence of critical data on IFA receipt among adolescents in India, findings of this study would be useful for current NIPI devised by the Indian government to mitigate Fe-deficiency anaemia burden. Multivariable results indicate that IFA receipt varies with age, area of residence, religion, mother’s education and state of residence, but these predictors affects males and female adolescents differently.

Overall, the coverage of IFA supplementation in Bihar and Uttar Pradesh was low, among both male and female adolescents. Low coverage of IFA would disproportionately affect female adolescents for their high Fe requirements lead to depleted Fe stores and a high prevalence of anaemia, and it could cause various adverse effects through their life course^(2)^. The poor performance in IFA supplementation in both states could be attributed to poor performance in healthcare services which is often associated with crisis of healthcare workers and lack of funding among other reasons^(13)^, and socio-economic characteristics of IFA beneficiaries play a critical role in IFA receipt.

While overall coverage remains low, an unacceptably low consumption of IFA (in last 1 month of the survey date) poses serious threat to the success of anaemia reduction programme in the state of Bihar and Uttar Pradesh. A careful interpretation of IFA consumption figure is needed, as adolescents who did not take IFA either did not have access to IFA or they did not consume IFA despite having it in their possession. Whatever the reasons of low intake of IFA are, one cannot deny the fact that low compliance with IFA consumption is rampant in India and earlier studies where consumption of IFA among adolescents was estimated to be very low for various reasons starting from forgetfulness to fear of side effects and lack of perceived benefits^(1,14)^.

Multivariate logistic regression analysis reveals that male adolescents aged 15–19 years were less likely to receive IFA. Recent evidence on sex-stratified analysis of IFA receipt among adolescents in India is rare, and most studies focus on women of reproductive age group and pregnant women. A recent study in West Bengal^(1)^ documented a lower prevalence of IFA receipt among adolescents, and this study calls for more research on IFA receipt among male adolescents. Adolescents living in rural area had higher odds of receiving IFA. Similar to this study, a recent study^(15)^ demonstrated that women living in rural were more likely to receive food supplement from AWC, and this trend could be attributed to government-focused nutrition intervention in rural area. However, the irony is, CNNS 2016–2018^(4)^ reported that adolescents in urban India had a higher prevalence of Fe deficiency compared to rural counterpart which calls for reinvestigation of distribution of IFA supplementation programme in urban India. Although it would be premature to comment if IFA intervention in rural India functions better than urban counterpart, but with some confidence the performance of IFA supplementation programme in urban Bihar and Uttar Pradesh may be questioned. Female adolescents belonged to Islam/other religion was less likely to receive IFA as compared to females who were followers of Hinduism, showing lower access of healthcare service utilisation among women followers of Islam/other religion^(15)^. Although the burden of anaemia is likely to be low among followers of Islam due to consumption of non-vegetarian diet among them, Islamic women tend to have low autonomy combined with low level of education which may prevent them from accessing IFA distribution programme. Mother’s education of female adolescents seems to have helped them receiving IFA, as compared to adolescents of uneducated mothers. This finding is consistent with earlier studies^(16–18)^ that empirically demonstrated various pathways about how maternal education could be a protective factor for strengthening the nutrition of their children.

Findings of this study should be interpreted by considering its few limitations. First, reporting on IFA receipt might not be free from recall errors and social desirability bias. Second, this study does not use an exhaustive list of potential predictors due to unavailibility of information on all aspects influencing performance of IFA supplementation. Third, the absence of detailed data on both receipt and (non-) consumption of the IFA supplement limits our understanding about the performance of NIPI. Finally, UDAYA survey collected information on IFA receipt preceding 1 year of survey date, which is not comparable with performance of weekly IFA supplementation. Despite these limitations, findings of this study will be a tremendous add-on asset for providing meaningful guidance for IFA intervention among adolescents in Bihar and Uttar Pradesh. This study calls for an intense intervention for increasing coverage of IFA receipt and consumption among adolescents across all socio-economic groups. The success of Anaemia Free Movement launched by Prime Minister of India is highly dependent on the success of the IFA supplementation programme^(19,20)^ in Bihar and Uttar Pradesh that houses over 72 million of India’s adolescents.
